# Maternal adjustment in expatriate and non-expatriate contexts: examining the role of sensory processing sensitivity and perceived social support

**DOI:** 10.3389/fpsyg.2025.1539885

**Published:** 2025-07-23

**Authors:** Kalimba Kinti Lagarrigue, Marta Sadurní Brugué, Bianca P. Acevedo, Moises Betancort

**Affiliations:** ^1^Doctoral Program in Psychology, Health and Quality of Life, University of Girona, Girona, Spain; ^2^Laboratory of Attachment and Human Development, University of Girona, Girona, Spain; ^3^Department of Psychological and Brain Sciences, University of California, Santa Barbara, Santa Barbara, CA, United States; ^4^Departamento de Psicología Clínica, Psicobiología y Metodología, Universidad de La Laguna, La Laguna, Spain

**Keywords:** expatriate mothers, sensory processing sensitivity, social support, maternal adjustment, differential susceptibility, low sensory threshold, ease of excitation, environmental sensitivity

## Abstract

**Background/purpose:**

This study examines maternal adjustment in the context of expatriation, where mothers face intensified cultural, social, and psychological challenges. Perceived social support, a key predictor of maternal well-being, may buffer these stressors. Sensory Processing Sensitivity (SPS)—a biological trait associated with heightened environmental and emotional sensitivity—may moderate the influence of maternal adjustment differently depending on the context (expatriation) and perceived social support. This study examines whether SPS and its subscales (Low Sensory Threshold [LST], Ease of Excitation [EOE], and Aesthetic Sensitivity [AES]) influence the link between perceived support and maternal adjustment differently among expatriate and non-expatriate mothers.

**Methods:**

Participants were 279 mothers, ages 20–49, with a mean of 1.9 children (SD = 1.2) aged 0–8 years. Mothers were recruited through targeted advertisements on social media, networks, and forums. Of these, 169 were expatriates, and 110 were non-expatriates. All participants completed measures of maternal adjustment (EMQ), perceived social support (MSPSS), and Sensory Processing Sensitivity (HSP-12 Scale) online.

**Results:**

Overall, SPS and its subdimensions (LST, EOE, and AES) were positively associated with maternal adjustment, with highly sensitive mothers reporting better adjustment than those with lower sensitivity levels. Among expatriate mothers, SPS was a significant predictor of maternal adjustment, and the interaction between SPS and social support significantly predicted maternal adjustment. Also, main effects were observed for each of the SPS subscales (AES, EOE, and LST), with AES and EOE also showing significant interactions with SPS in predicting maternal adjustment. No significant effects of SPS and support, or their interaction and were found for non-expatriate mothers.

**Conclusion:**

This study highlights the complex interplay between social support, SPS, and maternal adjustment, demonstrating that sensitivity shapes how mothers experience and benefit from social support. Overall, SPS was associated with better maternal adjustment. Also, in the context of expatriation (with additional challenges), SPS and its interaction with support were associated with better maternal adjustment. These findings suggest a positive outlook for mothers with high SPS, especially in contexts with social support. Also, they highlight the need for targeted interventions tailored to highly sensitive mothers, particularly those in expatriate contexts, to enhance maternal well-being and facilitate adjustment to motherhood.

## 1 Introduction

Expatriation is a complex and demanding experience that introduces significant psychological, social, and emotional challenges for individuals and their families. Unlike other forms of migration, which may be driven by economic necessity or forced displacement, expatriation is often characterized by structured, employer-driven relocations with a predetermined duration and varying degrees of organizational support (Fischlmayr and Puchmüller, 2015; Harari et al., 2018). While this structured nature may provide some logistical advantages, it does not shield expatriates from profound disruptions to their social networks, cultural dislocation, and heightened psychological stress (Brown, 2008). A key challenge in expatriation is the abrupt loss of established social and emotional support systems, including extended family, long-standing friendships, and familiar cultural frameworks that typically provide stability and reassurance. Additionally, expatriates must navigate the demands of adapting to a new cultural environment, which often involves language barriers, unfamiliar social norms, and a lack of immediate, trusted social connections.

It is well known that the transition to motherhood is compounded by significant psychological, social, and emotional challenges that require substantial adaptation (Stern and Bruschweiler-Stern, 1998). Access to broader networks, such as peer communities and structured social interactions, is linked to better maternal adaptation, highlighting the importance of both social and environmental factors (Greven et al., 2019; De Sousa Machado et al., 2020). Expatriation introduces additional complexities that can affect maternal adjustment, further emphasizing the need for systemic and structural support to help mothers navigate shifting self-concept, evolving personal and professional roles, and the challenges of maintaining well-being within unfamiliar social contexts (Neely and Reed, 2023).

Expatriation may disrupt access to established support networks, leaving mothers with fewer resources for emotional reassurance and practical assistance (Zeynalova, 2023). Additionally, expatriate mothers often carry the dual responsibility of adjusting to their new cultural environment while ensuring their children’s successful adaptation, a task further complicated when their partners are heavily committed to demanding professional obligations (Copeland and Norell, 2002). However, social support helps reduce stress, improve coping strategies, and enhance maternal well-being (Leahy-Warren et al., 2011; Figueiredo and Costa, 2009; Hijazi et al., 2021; Sharifipour et al., 2023).

Beyond social support and despite the significant challenges that motherhood entails, individual differences may also shape how mothers experience and respond to these challenges, whether in familiar or foreign environments (Taubman Ben-Ari et al., 2009). In this context, the biological trait of Sensory Processing Sensitivity (SPS)—which is associated with enhanced responsivity to the environment and others, as shown by enhanced empathy (e.g., Aron and Aron, 1997; Acevedo et al., 2014)— may explain individual differences in how mothers—both expatriate and non-expatriate—navigate their transition to motherhood. According to the theories of Differential Susceptibility and Environmental Sensitivity, highly sensitive individuals exhibit intensified reactions to both negative and positive contexts, making them more vulnerable to stressors, but also more receptive to supportive conditions (Belsky and Pluess, 2009).

In the context of motherhood, SPS can significantly influence maternal adjustment, as heightened emotional awareness, empathy, and deep processing of experiences shape both stress regulation and caregiving abilities (Acevedo et al., 2014; Aron et al., 2019). Highly sensitive mothers tend to be more emotionally attuned to their children, yet they may also experience increased difficulties in self-regulation when faced with challenges such as postpartum recovery, sleep deprivation, and the demands of infant care (Wu et al., 2021). In this vein, the literature shows that individuals with heightened SPS may experience increased stress and fatigue due to overstimulation in demanding environments (Gerstenberg, 2012). Additionally, they tend to exhibit increased emotional reactivity and introversion, which can influence their stress levels and coping mechanisms (Lionetti et al., 2018). This high sensitivity may increase maternal stress and fatigue, particularly in mothers with limited social support, as they may struggle to regulate their responses to overwhelming stimuli.

Building on this, research highlights that SPS is a multifaceted trait comprising distinct factors that differentially influence individual experiences. Low Sensory Threshold (LST), characterized by heightened reactivity to sensory input, makes individuals more sensitive to environmental stimuli, leading to increased discomfort in overstimulating conditions. Ease of Excitation (EOE) refers to how easily an individual becomes emotionally and physiologically aroused, which can result in higher emotional overload and difficulties in stress regulation (Pluess and Boniwell, 2015). These dimensions tend to amplify stress responses, making individuals more reactive to environmental demands.

In contrast, Aesthetic Sensitivity (AES) is associated with a heightened engagement with sensory and emotional experiences, which may contribute to deeper absorption of positive stimuli and potentially mitigate stress responses (Smolewska et al., 2006). It was noted that individuals with heightened aesthetic sensitivity exhibited greater levels of openness and agreeableness and were more inclined to use adaptive coping strategies (Chacón et al., 2024).

Thus, in the context of motherhood, SPS may shape maternal experiences in ways that are both demanding and enriching. Recognizing the complex effects of SPS is essential for developing personalized interventions, particularly for mothers facing additional stressors, such as those in expatriate contexts with reduced social support. A deeper understanding of how SPS interacts with environmental and social factors can inform the development of targeted support strategies that not only mitigate stressors but also harness the strengths of highly sensitive mothers (Bader et al., 2023), ultimately fostering better maternal well-being and adaptation.

## 2 Study aims and hypothesis

This study aims to investigate maternal adjustment in the context of expatriation, considering perceived social support and Sensory Processing Sensitivity (SPS) as key variables shaping this experience. Specifically, it explores how the perception of social support affects maternal adjustment and whether this relationship is moderated by individual differences in SPS. The expatriate versus non-expatriate context is examined across both aims to account for environmental variation in perceived support and maternal experience.

Aim 1 is to explore how perceived social support predicts maternal adjustment in mothers living in both expatriate and non-expatriate contexts.

Aim 2 is to examine whether SPS moderates the relationship between perceived social support and maternal adjustment, and whether this moderation differs depending on expatriation status.

From these aims, the following hypotheses are proposed:

*H1a:* Higher levels of perceived social support will be associated with better maternal adjustment across the sample, regardless of expatriate status. This hypothesis is based on literature demonstrating the protective effects of social support on maternal well-being (Leahy-Warren et al., 2011; Figueiredo and Costa, 2009; Figueiredo et al., 2008; Williamson et al., 2023).

*H1b:* Non-expatriate mothers will report higher levels of perceived social support and better maternal adjustment than expatriate mothers. Expatriation is associated with the disruption of familiar support systems and increased cultural adaptation demands, which can negatively impact well-being (Brown, 2008). In contrast, non-expatriate mothers typically retain more stable social networks, which may facilitate a smoother adjustment process.

*H2a:* SPS will moderate the relationship between perceived social support and maternal adjustment, such that highly sensitive mothers with high perceived support will report better maternal adjustment than those with low support. This aligns with the Differential Susceptibility theory, which posits that highly sensitive individuals are more responsive to environmental inputs, experiencing heightened benefit from supportive contexts (Belsky and Pluess, 2009).

*H2b:* The moderating effect of SPS will differ by expatriate status. Specifically, among expatriate mothers, those high in SPS and with high perceived social support will report significantly better adjustment than those with low support. In contrast, among non-expatriate mothers, this interaction is expected to be weaker or absent. Expatriation presents a unique constellation of stressors, and the combination of high SPS with environmental complexity may intensify mothers’ responsiveness to perceived support (Zeynalova, 2023).

## 3 Method

### 3.1 Participants

The study sample comprised 279 mothers recruited through targeted advertisements on social media platforms (Facebook, LinkedIn, Twitter), expatriate networking groups, and parenting forums. Before participation, mothers provided electronic informed consent, which outlined their rights, the voluntary nature of the study, and the option to withdraw at any time without consequences. No financial compensation was provided.

The whole survey was completed online, and it included demographic questions and standardized psychological measures assessing: maternal adjustment, Sensory Processing Sensitivity (SPS), and perceived social support. The estimated completion time was 30–45 min, given the inclusion of multiple questionnaires intended for future research publications.

Participants were 279 highly educated mothers, with 84.31% holding a graduate or postgraduate degree. The majority were married (87.95%) and employed full-time (74.72%). Most participants (89.2%) were 20–49 years of age. The mean number of children per household was 1.9 (SD = 1.2), and 95% of mothers had children between 0 and 8 years of age.

Participants were divided into two groups based on expatriate status: 169 expatriate mothers (60.57%) and 110 non-expatriate mothers (39.42%). Among expatriate participants, the most common countries of origin were the United States, Australia, South Africa, and Canada. At the time of participation, expatriate mothers were primarily residing in countries including the United States, Thailand, India, Lebanon, and Australia. Expatriate mothers relocated primarily for work-related reasons, often through employer-assigned expatriation programs that involved financial support, predefined job responsibilities, and a limited duration of stay (Fischlmayr and Puchmüller, 2015; Harari et al., 2018). Among expatriate participants, 46.48% originated from North America (Canada, United States), 29.50% from South America and Asia, and 24.02% from Europe, Africa, and Oceania. Non-expatriate mothers resided in their countries of origin, with 79.67% from Spain, Germany, Sweden, or the United Kingdom, and 20.33% from other European and non-European countries.

To participate in the study, mothers were required to have sufficient proficiency in English, as all questionnaires were administered in their original English versions without adaptations or translations. Participants who did not complete all questionnaires were excluded from the final analysis.

### 3.2 Procedure

The study protocol was reviewed and approved by the Ethics Committee of Universitat de Girona. Data were collected through a cross-sectional online survey, distributed via Qualtrics, which facilitated access to a geographically diverse sample.

### 3.3 Measures

Sociodemographic data were collected, including age, marital status, employment status, number of children, and expatriation status.

The Experience of Motherhood Questionnaire (EMQ): a questionnaire that assesses coping and emotional well-being in mothers with small children (Astbury, 1994). It consists of 20 items. Participants respond using a Likert-type scale ranging from “Not at all” (1) to “Very much so” (4), indicating their agreement or level of endorsement for each statement. The EMQ is structured around six factors: Maternal Anxiety/Concern, Coping with Baby, Personal Autonomy, Coping/Satisfaction with Life, Maternal Overload, and Extrinsic Support. A slight modification was made to the items of the Experience of Motherhood Questionnaire (EMQ), replacing the term “baby” with “child.” This adjustment was necessary to ensure the inclusion of mothers whose children were no longer infants, allowing for a broader representation of maternal experiences. Internal consistency was reported as 0.80.

The Highly Sensitive Person Scale (HSP-12): a 12-item scale that measures Sensory Processing Sensitivity (SPS). The scale includes five items on Ease of Excitation (EOE), three items on Low Sensory Threshold (LST), and four items on Aesthetic Sensitivity (AES). The scale was scored on a 7-point Likert scale (1 = not at all, 7 = extremely). Mean scores were calculated for the SPS total score, and the three subscales were analyzed separately to examine whether distinct aspects of sensitivity differentially moderated the relationship between social support and maternal adjustment. Internal consistency in this sample was high, with Cronbach’s alpha = 0.87 for the SPS total score; *α* = 0.84 for EOE, α = 0.63 for LST, and α = 0.75 for AES.

The Multidimensional Scale of Perceived Social Support (MSPSS): a self-report instrument designed to assess perceived social support from three primary sources: Family, Friends, and Significant Others. This 12-item questionnaire utilizes a Likert scale ranging from “Very Strongly Disagree” to “Very Strongly Agree.” Developed by Zimet et al. (1988), the MSPSS demonstrates robust factorial validity, indicating that its three subscales effectively capture distinct dimensions of perceived social support, along with good internal reliability. Internal consistency of this scale in this study was excellent, with Cronbach’s alpha = 0.97 for the total score.

### 3.4 Data analysis

All continuous variables were described using means and standard deviations. Preliminary associations among study variables were examined using Pearson correlations, and reliability assessments were conducted for all scales and subscales of interest.

Stepwise multiple regression analyses were conducted to examine whether SPS, total and subscale scores (EOE, LST, AES), moderated the relationship between perceived social support and maternal adjustment in a differential expatriate context (expatriate vs. non-expatriate mothers). For each stepwise regression, the following models were tested: (1) main effects, (2) two-way interactions: SPS total scores (or subscales) with perceived social support, and SPS with expatriate status, and (3) the three-way interaction of SPS, perceived social support, and expatriate status. The significance of incremental R-squared (*Δ*R2) was used to select the optimal mode. Assumptions for regression analyses, including linearity, homoscedasticity, normality of residuals, and multicollinearity, were assessed. Outliers were identified and examined to ensure that they did not unduly influence the results. Analyses were performed using Jamovi software (The Jamovi Project, 2024).

## 4 Results

### 4.1 Descriptive statistics

Descriptive statistics for all study variables are presented in Table 1. While most variables did not significantly differ based on expatriate status (ExpMom vs. NoExpMom), perceived social support was significantly lower among expatriate mothers (*p* < 0.05). Reliability analyses demonstrated good internal consistency for the SPS scale (both overall and subscales), comparable to previous empirical studies employing the HSP-12 scale.

**Table 1:** Descriptive measures for variables of interest by group. Cronbach alpha in parentheses for each scale and subscales.

Descriptives	Group	*N*	Mean	SD
SPS (0.87)	ExpMom	169	4.70	1.09
	NoExpMom	110	4.98	1.20
AES (0.75)	ExpMom	169	5.21	1.24
	NoExpMom	110	5.35	1.31
LST (0.63)	ExpMom	169	3.36	1.14
	NoExpMom	110	3.77	1.18
EOE (0.84)	ExpMom	169	5.54	1.64
	NoExpMom	110	5.82	1.75
MSPSS (0.97)	ExpMom	169	2.32	1.36
	NoExpMom	110	5.54	1.04
EMQ (0.80)	ExpMom	169	2.47	0.25
	NoExpMom	110	2.50	0,29

SPS, Sensory Processing Sensitivity total score; AES, Aesthetic Sensitivity; LST, Low Sensory Threshold; EOE, Ease of Excitation; MSPSS, Multidimensional Scale of Perceived Social Support total score; EMQ, Experience of Motherhood Questionnaire total score.

### 4.2 Correlation analysis

As a preliminary step, we explored bivariate correlations between the main variables of interest (see Table 2). These analyses revealed significant positive associations between SPS and maternal adjustment. All three SPS subdimensions—Ease of Excitation (EOE), Low Sensory Threshold (LST), and Aesthetic Sensitivity (AES)—were positively correlated with maternal adjustment. While these associations are not part of our primary aims or hypotheses, they provide valuable context for interpreting the moderation models.

**Table 2:** Correlation for variables of interest for entire sample.

Correlations among variables of interest								
	AES	LST	EOE	SPS	SIGOTHE	FAMI	FRIEND	MSPSS
AES	—							
LST	0.588***	—						
EOE	0.409***	0.604***	—					
SPS	0.773***	0.858***	0.852***	—				
SIGOTHE	0.010	0.161**	0.084	0.100	—			
FAMI	0.038	0.216***	0.070	0.123*	0.771***	—		
FRIEND	0.01	0.141*	0.062	0.079	0.790***	0.706***	—	
MSPSS	0.018	0.188**	0.079	0.110	0.938***	0.900***	0.906**	—
EMQ	0.254***	0.274***	0.233***	0.303***	0.070	0.052	0.029	0.056

SPS, Sensory Processing Sensitivity total score; AES, Aesthetic Sensitivity; LST, Low Sensory Threshold; EOE, Ease of Excitation; SIGOTHE, significant to others; FAMI, significant to family; FRIEND, significant to friends; MSPSS, Multidimensional Scale of Perceived Social Support total score; EMQ, Experience of Motherhood Questionnaire total score.

### 4.3 Regression analysis

To explore the moderating role of SPS in the relationship between perceived social support (MSPSS) and maternal adjustment (EMQ), a series of stepwise multiple regression analyses were conducted. Following the non-significant three-way interaction among SPS, MSPSS, and expatriate status, the sample was stratified by expatriate status. This allowed for an exploratory examination of two-way interaction models within each group—expatriate and non-expatriate mothers (see Table 3).

**Table 3:** Stepwise regression model for SPS total score, MSPSS and expatriate status predicting main effect and interaction over EMQ.

Model Coefficients–EMQ					
Model	Predictor	Estimate	SE	*t*	
Model 1	Intercept ᵃ	2.406	0.149	16.11**	
	SPS		0.01564	0.037	0.49
	MSPSS	−0.117	0.052	−2.22*	
	Expatriate Status	0.359	0.022	1.62	
Model 2	SPS ✻ MSPSS	0.023	0.010	2.13*	
	SPS ✻ Expatriate Status	−0.094	0.046	−2.03*	
Model 3	SPSTotal_R ✻ MSPSS ✻	0.005	0.005	0.93	

Δ*R*² Model 1 vs Model 2 = 1.98, *F* (2,273) = 3.053, *p* < 0.05. Model 2 vs. Model 3 = 0.88 (n.s.). Reference level ExpMom. **<0.01, *<0.05.

**Figure 1:**
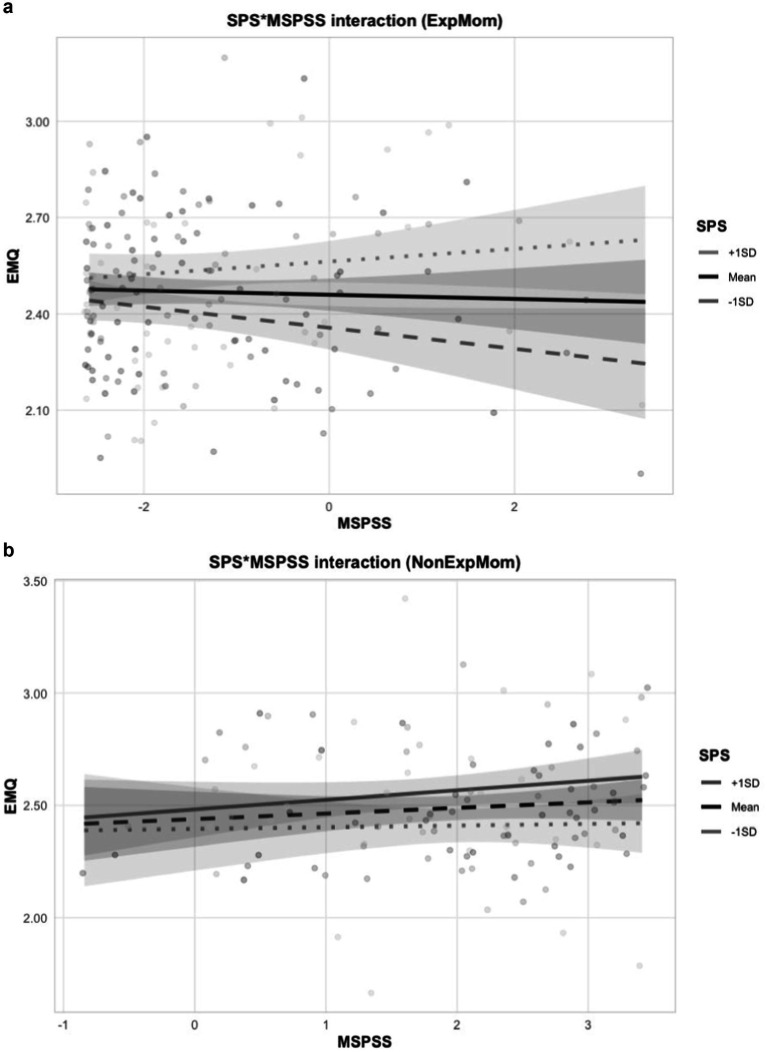
Interaction plot for SPS*MSPSS on Expatriate Mothers **(a)** and Non Expatriate Mothers **(b)**. The Johnson-Neyman Interval test showed that when SPS is outside the interval [−1.12, 3.29], slope of MSPSS is *p* < 0.05. SPS range is [−3.06, 2.19].

In the expatriate group, SPS was a significant predictor of maternal adjustment. Specifically, Model 1 indicated a significant positive main effect of SPS on EMQ (*β* = 0.071, SE = 0.017, *t* = 4.10, *p* < 0.05), whereas MSPSS did not contribute significantly. In Model 2, the interaction between SPS and MSPSS was significant (*β* = 0.026, SE = 0.011, *t* = 2.30, *p* < 0.05) (see Figure 1). Additionally, a significant main effect of MSPSS was observed in this model (*β* = −0.133, SE = 0.055, *t* = −2.38, *p* < 0.05).

In contrast, in the non-expatriate group, neither main effects nor interaction effects reached statistical significance.

#### 4.3.1 Moderation by SPS subscales

To further examine differential patterns of sensitivity, additional stepwise regression analyses were performed separately for each SPS subscale—Low Sensory Threshold (LST), Aesthetic Sensitivity (AES), and Ease of Excitation (EOE)—within each expatriation group.

##### 4.3.1.1 Expatriate mothers

- LST: Model 1 revealed a significant main effect of LST on maternal adjustment (*β* = 0.06, SE = 0.01, *t* = 3.60, *p* < 0.05). However, in Model 2, no significant interaction between LST and MSPSS was observed, nor were additional main effects significant.
- AES: A significant main effect was observed in Model 1 (*β* = 0.053, SE = 0.01, *t* = 3.47, *p* < 0.05). In Model 2, MSPSS approached significance (*β* = −0.09, SE = 0.04, *t* = −1.93, *p* = 0.054), and the interaction between AES and MSPSS was significant (*β* = 0.01, SE = 0.008, *t* = 2.06, *p* < 0.05), indicating a moderating effect of AES on the relationship between support and adjustment.
- EOE: A significant main effect of EOE was observed (Model 1: *β* = 0.034, SE = 0.011, *t* = 2.93, *p* < 0.05). In Model 2, a significant interaction emerged between EOE and MSPSS (*β* = 0.02, SE = 0.008, *t* = 2.15, *p* < 0.05).

##### 4.3.1.2 Non-expatriate mothers

In the non-expatriate group, analyses for LST, AES, and EOE subscales yielded no statistically significant main or interaction effects.

## 5 Discussion

This study investigated maternal adjustment in the context of expatriation, focusing on the role of perceived social support and SPS in shaping maternal adjustment. Rather than examining SPS as uniformly beneficial or detrimental, our findings suggest that higher sensitivity is associated with better maternal adjustment when mothers perceive social support. This is consistent with SPS theory suggesting that SPS is associated with greater depth of processing, awareness of subtleties in the environment, a tendency to become overstimulated, stronger emotional responses (both positive and negative), and empathy to others’ affective cues (e.g., Aron and Aron, 1997; Wolf et al., 2008; Acevedo et al., 2018). Also, our findings are consistent with neuroimaging research showing that SPS is associated with stronger empathy-related neural activity in response to a loved one or strangers’ happy or sad facial expressions, as well as higher response in the brain’s reward regions in response to face images of a spouse smiling (Acevedo et al., 2014).

Our findings further revealed that the association between SPS and maternal adjustment varies as a function of environmental context and its interaction with perceived social support, particularly among mothers in expatriate settings. This aligns with the frameworks of Differential Susceptibility and Environmental Sensitivity, which propose that highly sensitive individuals exhibit amplified responses to both adverse and supportive conditions (Belsky and Pluess, 2009; Pluess, 2015; Aron et al., 2012). Consistent with our hypothesis H2a, SPS moderated the relationship between perceived social support and maternal adjustment, such that expatriate mothers with higher levels of SPS reported better adjustment when they also perceived strong social support. In line with hypothesis H2b, this moderating effect was specific to the expatriate group and not observed among non-expatriate mothers, suggesting that the regulatory impact of SPS is particularly salient under more challenging environmental conditions. Moreover, Aesthetic Sensitivity (AES) emerged as a central moderator in the relationship between support and adjustment. The amplification effect observed here may reflect a form of sensory-affective attunement that enhances the internalization of supportive experiences, contributing to improved maternal well-being. In the context of motherhood, this capacity to register and integrate positive environmental input may foster a more resilient, meaning-oriented adaptation process.

This interpretation aligns with research identifying AES as a perceptual lens attuned to subtle emotional and aesthetic cues, linked to trait absorption, affective richness, and openness to beauty and coherence (Smolewska et al., 2006). Within the vantage sensitivity framework (Pluess and Belsky, 2013), AES may function as a facilitating trait, allowing highly sensitive individuals to derive disproportionate benefit from enriched or supportive environments. In expatriate motherhood, this may include the ability to find resonance and meaning in new cultural surroundings, offering alternative sources of comfort and coherence.

EOE also moderated the relationship between social support and maternal adjustment. Among expatriate mothers, higher levels of SPS and perceived support were associated with enhanced maternal adjustment, indicating that social resources may serve as a key buffer for individuals whose heightened emotional reactivity would otherwise increase vulnerability to environmental stressors.

While H1a anticipated a general positive association between perceived social support and maternal adjustment, our findings suggest that this relationship is contingent on individual differences, such as SPS in general, highlighting that support may function not only as a protective factor, but as a regulatory mechanism that helps manage affective overload in highly sensitive individuals navigating the complexities of relocation and acculturation. This was supported by the significant interaction between EOE and social support, resulting in better maternal adjustment. Given this focus on internal experiences, self-report methods were central to capturing the lived experience of adjustment. However, future research may complement these with observational or clinical assessments to enrich our understanding of behavioral adaptation.

Further, expatriate mothers with higher LST levels demonstrated increased maternal adjustment, although the interaction with social support was not significant. While LST has been associated with heightened stress reactivity, our findings suggest that this reactivity can be redirected toward adaptive responsiveness, notably among expatriate mothers.

Together, these findings provide a more granular understanding of how SPS operates in maternal adjustment. Notably, while social support was conceptualized as a universally beneficial predictor of maternal adjustment (H1a), our results indicate that its effects are more conditional than originally hypothesized. The absence of a significant main effect suggests that perceived support alone does not predict adjustment uniformly but may require specific individual or contextual configurations, such as high SPS or the presence of expatriation-related stress, to become salient. Similarly, H1b, which anticipated better adjustment and higher perceived support among non-expatriate mothers, was only partially supported. Although non-expatriate mothers did report greater perceived support, this did not translate into significantly higher maternal adjustment.

Highly sensitive mothers appear to experience motherhood in a qualitatively distinct way, suggesting better adjustment, even in challenging circumstances, such as expatriation. Expatriation, far from being a uniform stressor, may offer both risk and opportunity depending on how it aligns with sensitivity. Structured expatriate communities, cultural novelty, and imposed routines may facilitate a balance between sensory engagement and adaptive retreat, allowing some highly sensitive mothers to thrive.

This perspective moves beyond the notion of SPS as either a vulnerability or a protective factor. It instead highlights the importance of identifying how specific environmental and social features interact with different SPS subdimensions. The aim should not be to categorize sensitivity but to understand its context-dependency, to better adapt external conditions to support well-being and promote functional adjustment. It is important to consider that these interpretations arise within a specific demographic context. These findings should also be interpreted within the specific demographic profile of the sample, composed predominantly of highly educated mothers, a characteristic typical of structured expatriate populations.

## 6 Novelty of this paper

This study offers a novel contribution to the literature by integrating three previously distinct domains—SPS (SPS), perceived social support, and maternal adjustment—within the underexplored context of expatriate motherhood. While prior research has examined SPS in relation to parenting, child attunement, and caregiving behaviours, this study shifts focus to maternal adjustment itself, addressing a critical gap in how sensitivity traits shape the experience of becoming a mother under complex environmental conditions.

A key innovation lies in the fine-grained analysis of SPS subdimensions—specifically, the moderating roles of Aesthetic Sensitivity (AES), Ease of Excitation (EOE), and Low Sensory Threshold (LST)—in shaping how perceived support influences maternal adaptation. Rather than treating SPS as a monolithic trait, this study empirically demonstrates how its components function differently across expatriate and non-expatriate contexts. In particular, the finding that AES amplifies the adaptive role of perceived support introduces a nuanced perspective on how sensory-affective attunement may support meaning-making and psychological resilience during the transition to motherhood.

Moreover, the identification of context-specific effects, such as the interaction between EOE and social support among expatriate mothers, advances a more dynamic model of adjustment. These patterns challenge traditional direct-effect models of support by showing that the impact of perceived support depends not only on its availability but on how it is interpreted and integrated by individuals with distinct sensory-affective profiles. This opens new directions for conceptualising support not simply as a protective resource, but as a variable processed through perceptual, emotional, and contextual filters.

By investigating the intersection of sensitivity, environment, and perceived support, this study introduces a context-sensitive framework for understanding maternal adjustment. Rather than isolating vulnerability or resilience as fixed traits, the findings emphasise the need to examine how specific environmental configurations interact with individual sensitivity dimensions. In doing so, the study offers both theoretical advancement and practical insight, supporting the development of tailored interventions that consider how highly sensitive mothers engage with their environments, particularly during transitional life phases like expatriation.

Given that SPS characterises a substantial portion of the population, identifying the environmental and relational conditions that enhance or hinder adjustment among highly sensitive individuals is vital. This research thus lays the groundwork for developing nuanced, evidence-informed strategies to promote maternal well-being in culturally and emotionally complex settings.

## 7 Limitations and future directions

Several limitations should be acknowledged in interpreting the findings of this study. First, although the use of the Experience of Motherhood Questionnaire (EMQ) with SPS represents a novel approach, only the total EMQ score was analyzed. This precluded a more detailed exploration of its subscales, which may have provided greater insight into how specific aspects of maternal adjustment relate to sensitivity traits such as Low Sensory Threshold (LST). Future studies would benefit from disaggregating the EMQ to examine whether particular dimensions of maternal adjustment are differentially affected across expatriate and non-expatriate mothers.

Second, the heterogeneity of the sample—both within and across groups—introduces interpretive complexity, particularly regarding the role of cultural context in shaping self-reported experiences. Although the inclusion of a culturally diverse sample was an intentional strength of the design, especially for capturing the variability of expatriate motherhood, it also raises the possibility that cross-cultural differences in emotional expression, sensitivity processing, or perceived support may influence responses. Prior research suggests that individual traits such as SPS can be expressed or interpreted differently across sociocultural environments (Aron et al., 2010). Nonetheless, the measures used in this study are widely validated across cultures and underpin key findings in international SPS research. Future culturally grounded studies may still reveal subtle differences in how sensitivity and support are perceived.

Third, the cross-sectional design of the study limits inferences about developmental or temporal processes. Longitudinal research is needed to investigate how the relationships identified here—between SPS, perceived social support, and maternal adjustment—evolve over time, particularly during key stages of the motherhood transition and in response to environmental shifts.

Fourth, although variables such as time abroad and language proficiency were collected, they were not included in the current analyses, as they apply only to expatriate mothers and do not support valid cross-group comparisons. This study was designed to examine maternal adjustment through psychological constructs measurable across both expatriate and non-expatriate groups. However, certain contextual variables—such as duration of residence abroad, language proficiency, and perceived partner support—have been identified in previous expatriation research as particularly relevant to adjustment processes. These variables were therefore collected specifically for the expatriate subsample, where they hold theoretical and practical significance. Given that they are not applicable to non-expatriate mothers, they were excluded from the present comparative analyses. A follow-up study, currently in preparation, will examine these variables within the expatriate group to better understand how such contextual factors shape maternal adjustment in the absence of broader social support networks.

Fifth, because the study centers on maternal adjustment, its focus is deliberately restricted to mothers’ subjective experiences, perceptions of sensitivity, and perceived social support. Future research could usefully adopt a dyadic or relational lens to examine how maternal and partner dynamics interact in shaping adjustment. Similarly, while self-report methods were appropriate given the internal and perceptual nature of the constructs assessed, observational or clinician-based assessments could complement these data in future research, enriching our understanding of behavioral dimensions of maternal well-being.

Finally, the use of online recruitment methods through social media and expatriate networks may have introduced selection bias by underrepresenting mothers who are less digitally connected or socially engaged. While these platforms enabled access to a demographically relevant and globally distributed sample, future studies may consider additional recruitment strategies to reach more structurally marginalized or isolated groups.

## 8 Conclusion

This study contributes to a more differentiated understanding of how SPS shapes maternal adjustment, particularly within the complex context of expatriation. Rather than functioning solely as a moderator, SPS appears to define distinct pathways through which mothers experience and respond to the transition to motherhood. The role of specific SPS subdimensions—especially Aesthetic Sensitivity (AES), Ease of Excitation (EOE), and Low Sensory Threshold (LST)—underscores the need to consider sensitivity as a multifaceted construct that mediates the interpretation of, and adaptation to, environmental and social conditions.

Expatriation, in turn, emerges not as a uniformly adverse or beneficial factor, but as a contextual amplifier that may either support or strain these sensitivity-based processes. These findings suggest that maternal adjustment is shaped by the dynamic interplay between environmental configuration and individual sensory-affective traits.

By moving beyond static models of vulnerability or resilience, this study opens new perspectives on how maternal well-being can be supported through the design of context-sensitive environments and interventions that acknowledge sensory diversity. Future work should continue to examine how different SPS profiles interact with emotional regulation strategies and environmental adaptation over time, to promote more responsive and inclusive support frameworks for mothers across cultural settings.

## Data Availability

The datasets used in the current study are available at the OSF repository: https://osf.io/s5kyw/.
